# *Notes from the Field: *Norovirus Outbreaks Reported Through NoroSTAT — 12 States, August 2012–July 2022

**DOI:** 10.15585/mmwr.mm7138a3

**Published:** 2022-09-23

**Authors:** Anita K. Kambhampati, Mary E. Wikswo, Leslie Barclay, Jan Vinjé, Sara A. Mirza, NoroSTAT Network, NoroSTAT Network, Esther Rei, Brandon Sabina, Jennifer Beggs, Diana K. Riner, Elizabeth Cebelinski, Amy Saupe, Amanda Bartling, Brianna K.D. Loeck, Nicole Chase, Jessica Houston, Eric Brandt, Ellen Salehi, Emilio DeBess, Laura Tsaknaridis, Gregory Goodwin, Hani Mohamed, Mugdha Golwalkar, Linda Thomas, Mary Kathryne Donnelly, Haley Greene, Timothy Davis, Lynn Roberts, Rob Christensen, Matthew Peterson

**Affiliations:** 1 Division of Viral Diseases, National Center for Immunization and Respiratory Diseases, CDC.; Massachusetts Department of Public Health; Massachusetts Department of Public Health; Michigan Department of Health and Human Services; Michigan Department of Health and Human Services Bureau of Laboratories; Minnesota Department of Health; Minnesota Department of Health; Nebraska Public Health Laboratory; Nebraska Department of Health and Human Services; New Mexico Department of Health; New Mexico Department of Health; Ohio Department of Health; Ohio Department of Health; Oregon Health Authority; Oregon State Public Health Laboratory; South Carolina Department of Health and Environmental Control Public Health Laboratory; South Carolina Department of Health and Environmental Control; Tennessee Department of Health; Tennessee Department of Health Laboratory Services; Virginia Division of Consolidated Laboratory Services; Virginia Department of Health; Wisconsin State Laboratory of Hygiene; Wisconsin Department of Health Services; Wyoming Public Health Laboratory; Wyoming Department of Health

Norovirus is the leading cause of acute gastroenteritis in the United States (*1*). In April 2020, the incidence of norovirus outbreaks in the United States declined substantially, likely because of implementation of COVID-19–related nonpharmaceutical interventions, such as facility closures, social distancing, and increased hand hygiene (*2*). Similar declines were observed in other countries (*3*,*4*). Norovirus outbreaks in the United States increased rapidly starting in January 2022, approaching prepandemic (i.e., 2012–2019) levels. Norovirus transmission can be prevented by thorough handwashing and proper cleaning and disinfection of contaminated surfaces.

In 2012, CDC established the Norovirus Sentinel Testing and Tracking Network (NoroSTAT) to improve timeliness and completeness of surveillance for norovirus outbreaks that occur in the United States. NoroSTAT is a collaboration between CDC and 12 state health departments.* Outbreaks are defined as two or more cases of illness associated with a common exposure. NoroSTAT-participating states report a minimum set of data elements^†^ to the National Outbreak Reporting System^§^ for all confirmed norovirus outbreaks (i.e., outbreaks with two or more laboratory-confirmed norovirus cases) and suspected norovirus outbreaks (i.e., outbreaks with fewer than two laboratory-confirmed norovirus cases) within 7 business days of notification. These states also upload typing information for norovirus-positive outbreak specimens to CaliciNet,^¶^ the national norovirus laboratory surveillance network, within 7 business days of receipt of two outbreak-associated norovirus-positive stool specimens at the respective state public health laboratory. Outbreak reports are organized into surveillance years (i.e., August 1–July 31) based on the state funding cycle.**

During the 2021–2022 surveillance year (August 1, 2021–July 31, 2022), the 12 NoroSTAT-participating states reported 992 norovirus outbreaks to CDC (Figure). In comparison, the same states reported 1,056 and 343 norovirus outbreaks during the 2019–2020 and 2020–2021 surveillance years, respectively. The number of norovirus outbreaks reported by these states during prepandemic surveillance years ranged from 1,219 (2015–2016) to 1,471 (2018–2019). Norovirus outbreak characteristics reported by NoroSTAT-participating states during 2021–2022 were similar to those reported during prepandemic years. Most outbreaks (82%) were due to person-to-person spread (prepandemic range = 71%–85%). The majority (59%) of outbreaks occurred in long-term care facilities (prepandemic range = 53%–68%); 17% were laboratory-confirmed (prepandemic range = 22%–48%). Among laboratory-confirmed outbreaks with typing information during 2021–2022, a total of 43% were GII.4 Sydney(P16), which has been the predominant norovirus strain since its emergence during 2015–2016 (*5*).

**FIGURE:**
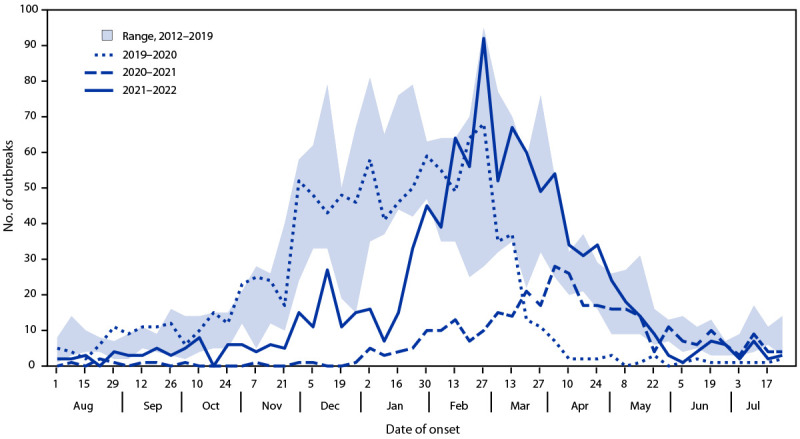
Number of norovirus outbreaks reported to the National Outbreak Reporting System by Norovirus Sentinel Testing and Tracking Network states, by month of outbreak onset — 12 states, 2012–2022*^,†,§,¶^ **Abbreviation:** NoroSTAT = Norovirus Sentinel Testing and Tracking Network. * Michigan and South Carolina joined the NoroSTAT network at the start of the 2015–2016 surveillance year; 2012–2015 data from these states were added for comparison. ^†^ Massachusetts and Virginia joined the NoroSTAT network at the start of the 2016–2017 surveillance year; 2012–2016 data from these states were added for comparison. ^§^ New Mexico and Wyoming joined the NoroSTAT network at the start of the 2018–2019 surveillance year; 2012–2018 data from these states were added for comparison. ^¶^ Nebraska joined the NoroSTAT network at the start of the 2019–2020 surveillance year; 2012–2019 data from this state were added for comparison.

The number of norovirus outbreaks that NoroSTAT-participating states reported during the 2021–2022 surveillance year was nearly three times the number reported during the 2020–2021 surveillance year. Nonpharmaceutical interventions implemented during the COVID-19 pandemic were likely effective in preventing outbreaks of other infectious diseases, including norovirus. As the use of nonpharmaceutical interventions has relaxed, norovirus outbreak incidence has returned to levels similar to those during prepandemic surveillance years, and GII.4 viruses continue to cause the largest proportion of norovirus outbreaks. Norovirus transmission can be prevented by handwashing thoroughly with soap and water, avoiding food preparation until ≥48 hours after symptoms end, and proper cleaning and disinfection of surfaces contaminated by vomitus or diarrhea.^††^

## Footnotes

* Massachusetts, Michigan, Minnesota, Nebraska, New Mexico, Ohio, Oregon, South Carolina, Tennessee, Virginia, Wisconsin, and Wyoming.

† Within 7 business days of health department notification, NoroSTAT-participating states are required to report the following data elements to CDC for all norovirus outbreaks: state report ID, primary mode of outbreak transmission, date of first illness onset (at the time of initial report), date of notification to state health department, state in which the outbreak exposure occurred, estimated total number of primary ill cases, confirmed or suspected etiology, and outbreak setting.

§
https://www.cdc.gov/nors/index.html

¶
https://www.cdc.gov/norovirus/reporting/calicinet/index.html

**
https://www.cdc.gov/ncezid/dpei/epidemiology-laboratory-capacity.html

††
https://www.cdc.gov/norovirus/about/prevention.html
